# Discussing dissection in anatomy education

**DOI:** 10.1007/s40037-015-0207-7

**Published:** 2015-09-10

**Authors:** Esther M. Bergman

**Affiliations:** Department of Educational Development and Research, Faculty of Health, Medicine and Life Sciences, Maastricht University, PO Box 616, 6200 MD Maastricht, The Netherlands

In this issue, Patel et al. [1] ask the question whether dissection is the only way to learn anatomy. The authors discuss some of the methods that students at a non-dissection based medical school have found beneficial to gain anatomical knowledge. In particular, they focus on the use of anatomy videos on YouTube, virtual models and the teaching of anatomy by surgeons in theatre. Exploring other options is not far-fetched when considering the space, time and money required to maintain a dissection room, and the problems many medical schools around the world encounter when trying to obtain enough cadavers. The paper by Patel et al. [1] is another contribution to the ongoing debate about cadaver dissection as an educational tool. I agree that cadaver dissection is indeed not the only way to teach and learn anatomy, but that does not mean we should disregard it.

Review studies have compared dissection to the use of prosected specimens (in which students can study whole or parts of cadavers dissected by anatomy staff in advance) [2] and other teaching tools [3]. Although not straightforward, the results seem to be slightly in favour of dissection. However, research has also shown that a dissection course is not a uniform learning experience [4]. Different students have different approaches in dealing with dissection, therefore undergoing divergent learning experiences, which may result in differences in the amount and form of knowledge between individuals. As this possibly applies to all learning methods, it should not come as a surprise that a combination of methods to learn anatomy gives the best results. An example is the study by Biasutto et al. [5] who found better results for students who only dissected cadavers compared with students who only used computer resources, but reported the best scores for the group of students who learned by both dissection and the use of computer resources. In light of these results, I personally feel that we need to answer the *when*, *what* and *how* of anatomy education.

*When is the best moment to study anatomy in the dissection room?* A recent study seems to indicate that anatomy education in the dissection room is most useful in the second of three steps of learning anatomy [6]. First, students can learn the vocabulary of anatomy (i.e. the names of the structures) through lectures or e-learning. Second, studying and understanding the 3D relationships of structures is best done in the dissection room, at least until 3D software and glasses become mainstream. The third step, discussing how anatomical knowledge can be used to explain signs and symptoms, can be done in settings such as tutorial groups. Furthermore, advocates of the use of dissection in undergraduate education frequently mention the (extra) opportunity for students to work on additional learning objectives, such as professionalism, manual dexterity, teamwork, self- and peer-evaluation, ethics etc. (e.g. Gregory et al. [7]).

Norman [8] has suggested that most medical specialists who rarely get to see inside the body may well get by with studying simplified anatomy, e.g. with schematics in textbooks and plastic life-like models, and may even benefit from such an approach. This idea may contribute to the interesting fact that while cadaver dissection seems to be on the way out in undergraduate education, it is gaining popularity in postgraduate education. Next to acquiring specific in-depth anatomical knowledge on the region(s) of interest, the dissection room is widely used to practice technical skills such as surgical or anaesthesiological approaches, performing an endoscopy, laparoscopy, biopsy, ultrasound etc. (e.g. Kshettry et al. [9]). I think the future will show us that postgraduate education is an (additional) appropriate moment to study anatomy in the dissection room.

*What anatomical knowledge can be studied with which learning method?* While less complex structures (e.g. abdominal organs) can be studied from a textbook or cadaver material, it is possible that students may get a better understanding of more complex anatomical structures (e.g. bones of the skull, the brain, course of a blood vessel) from virtual models. However, virtual models that have all the advantageous functions as described by Patel et al. [1] are not always instantly easy to navigate and need high-quality computers to run smoothly. In addition, the costs to obtain a license which makes a virtual model available to a large number of students can become very high. These issues may inhibit the full or partial substitution of cadaver dissection by virtual models in medical schools in certain countries.

In contrast, YouTube is accessible without charge around the world. Although using videos may be a very popular learning method and certainly has a lot of benefits [1, 10], Azer [11] and Raikos and Waidyasekara [12] have shown that YouTube is currently an inadequate source of information for learning at least surface anatomy and heart anatomy. Both studies looked at more than 200 uploaded videos, and found that only around 25 % of them provided adequate anatomical content. With over 50,000 hits in the first search for heart anatomy videos, and the absence of videos covering surface anatomy of, among others, the head and neck region, some students might perceive using YouTube as challenging, time consuming and frustrating as other students perceive cadaver dissection to be. When considering YouTube as an alternative or additional learning method, it seems indispensable that the involved faculty guides students in the search and selection of the best resources.

*How should anatomical knowledge be taught?* After decades of debate about cadaver dissection as a learning method, I feel we can conclude that there is no single method that can function as an answer for how anatomy should be taught. In my opinion it is not about the method you are using, but about how you are using it. Strategies such as increasing ‘time on task’, creating occasions for repetition and scaffolding (constructive learning), including opportunities for interaction between learners (collaborative learning) and placing learning in a context (contextual learning) are crucial when it comes to knowledge acquisition and retention [13]. The circumstances in which teaching methods are utilized are imperative, and fortunately these learning principles can be applied independently from any teaching method. Furthermore, the effect of a teaching method is often connected to the guidance students receive during their learning. Learning anatomy may benefit from ‘directed self-learning’ (in contrast to self-directed learning), suggesting self-study should be guided by experts in the subjects of both anatomy and medicine [14]. Because insufficient or excessive support can hamper a learning process, it is critical to determine the right type and amount of support (teaching methods) and guidance (presence and input of a teacher) and to fade at the appropriate time and rate [15].

It is true that in the past, lectures and cadaver dissection by students themselves was seen as essential to anatomy learning, and that anatomists and clinicians worry about the detrimental effect on students’ knowledge when removing the time-honoured method of cadaver dissection from anatomy education. However, complaints about declining anatomical knowledge were also heard before this process started [16]. Furthermore, students seem to be insecure about their anatomical knowledge, and keen to augment their knowledge using any learning method, regardless of the way they are taught [17, 18]. Although opinions may differ as to its scope, there is a general consensus that medical students definitely cannot do without anatomical knowledge, and consequently without anatomy education. However, in medical education today, the key question regarding anatomy education increasingly focuses on how education can be made as effective as possible. Therefore, it is important that future research investigates what and how students learn from dissection and other teaching methods, in order to provide teachers (and students) with information on which they can base their choices.
